# Systematic assessment of the medical utility of radiology and diagnostic Artificial Intelligence in fracture detection (SAMURAI-fracture): a protocol for a multicentre cluster-randomised controlled trial

**DOI:** 10.1136/bmjopen-2026-120631

**Published:** 2026-08-21

**Authors:** Alex Novak, James Vaz, Abdala T Espinosa Morgado, Sally Beer, Katrina Nash, Ashar Asif, Michael Lundemann, David Metcalfe, Matthew L Costa, Nick Woznitza, Mamta Bajre, Niks Kolosnicins, David Clarke, Rose Kunnath, Ammar Salem, Sachin Mandalia, Andrew G Murchison, Susan Cheng Shelmerdine, David Lowe, Jason Oke, Sarim Ather

**Affiliations:** 1 Oxford Clinical Artificial Intelligence Research (OxCAIR), Oxford, UK; 2 Emergency Medicine Research Oxford (EMROx), Oxford University Hospitals NHS Foundation Trust, Oxford, Oxfordshire, UK; 3 Oxford University Clinical Undergraduate School (OUCAGs), University of Oxford, Oxford, England, UK; 4 Radiobotics ApS, Copenhagen, Denmark; 5 Emergency Department, Oxford University Hospitals NHS Foundation Trust, Oxford, UK; 6 Nuffield Department of Orthopaedics, Rheumatology & Musculoskeletal Sciences, University of Oxford, Oxford, UK; 7 Kadoorie Institute for Trauma, Emergency and Critical Care, University of Oxford, Oxford, Oxfordshire, UK; 8 University College London Hospitals NHS Foundation Trust, London, England, UK; 9 University College London, London, England, UK; 10 Health Innovation Oxford and Thames Valley (HIOTV), Oxford, UK; 11 PPI Representative, Oxford, UK; 12 Royal Berkshire NHS Foundation Trust, Reading, England, UK; 13 Buckinghamshire Healthcare NHS Trust, Amersham, England, UK; 14 Oxford Health NHS Foundation Trust, Oxford, England, UK; 15 Clinical Radiology, Great Ormond Street Hospital for Children, London, UK; 16 UCL Great Ormond Street Institute of Child Health, London, UK; 17 NIHR Great Ormond Street Hospital Biomedical Research Centre, London, UK; 18 Digital Health Validation Lab, University of Glasgow, Glasgow, Scotland, UK; 19 Emergency Department, NHS Greater Glasgow and Clyde, Glasgow, Scotland, UK; 20 Oxford Biostatistics Ltd, Oxford, UK

**Keywords:** Fractures, Bone, Emergency Service, Hospital, Artificial Intelligence, RADIOLOGY & IMAGING

## Abstract

**Introduction:**

Fracture misdiagnosis is a common diagnostic error in emergency departments (EDs) and minor injury units (MIUs), leading to poor patient outcomes, unnecessary treatments and significant healthcare costs. Artificial intelligence (AI)-assisted fracture detection tools are now available for use in radiology workflows; however, the impact of these technologies on patient outcomes, experiences and overall care pathways in the real-world clinical setting is limited.

**Methods and analysis:**

We will conduct a prospective cluster randomised cross-over trial over a 6-month period, assessing the impact of an AI-assisted fracture detection tool in EDs and MIUs across 4 healthcare Trusts in the UK. Patients aged over 2 years old undergoing a plain film radiography for a suspected fracture as part of routine clinical care will be eligible for study enrolment. The trial will deploy Radiobotics’ RBfracture, a CE-approved AI-assisted medical device software for fracture detection at each site for 6 months. Randomisation will be at a cluster-level; a site will be randomised to begin with ‘AI on’ or ‘AI off’ for a month, followed by alternating active status each month for the remaining 5 months. The primary outcome will evaluate the incidence of ‘inappropriate healthcare contacts’ among patients receiving imaging for suspected fractures. This composite metric encompasses inappropriate referrals to fracture clinics, repeated hospital attendances and subsequent follow-up communications regarding missed fractures. The rates of these measures will be compared in the ‘AI on’ versus ‘AI off’ stages. Secondary outcomes will include patient-reported outcomes, clinician surveys, a predefined health economic evaluation assessing cost-effectiveness and budget impact and the diagnostic performance of the algorithm.

**Ethics and dissemination:**

The study has received ethical approval from the South Central—Oxford Research Ethics Committee (Reference: 25/SC/0252, approval date: 23 October 2025), and subsequently from the Health Research Authority (IRAS 3 57 391-A). The results of this study will be presented at relevant scientific conferences and peer-reviewed publications will be disseminated on wider public forums such as traditional and social media.

**Registration details:**

This is an ongoing, actively recruiting trial (ISRCTN23087950). Recruitment began in January 2026 and will run for 6 months at each site, with per-patient follow-up of 30 days and subsequent data analysis. This manuscript describes protocol version 1.1. The full protocol and statistical analysis plan are available from the corresponding author on request and will be deposited on the study’s public repository (https://github.com/Jason-L-Oke/SAMURAI) prior to publication of results.

**Trial registration number:**

ISRCTN23087950.

**STRENGTHS AND LIMITATIONS OF THIS STUDY**

- Systematic Assessment of the Medical Utility of Radiology and Diagnostic Artificial Intelligence-Fracture will be the first randomised controlled trial globally to measure the clinical and health economic impact of artificial intelligence (AI)-assisted fracture detection in emergency care.
- Involving four distinct National Health Service Trusts provides a geographically and demographically heterogeneous patient population to evaluate the intervention.
- The cross-over study design allows for each site to experience both AI and non-AI assisted pathways multiple times, reducing bias and the effects of confounders, and allowing within-site comparisons of outcomes.
- The study will directly address National Institute of Health and Care Research Early Value Assessment evidence generation requirements for artificial intelligence assisted fracture detection.
- This evaluation is limited to a single proprietary tool which will secure the generalisability of the study findings to other AI algorithms.

## Introduction

 Fracture misdiagnoses are the most common diagnostic errors in emergency departments (EDs), and the leading cause of successful legal claims related to ED care.^1 2^ In the UK, 1.8 million fractures occur annually with a lifetime prevalence nearing 40% and representing 5.1% of the £2.1 billion annual UK ED attendance costs.^3 4^

EDs are high-volume referrers of X-rays, with 2.1 million performed every year in England and estimates suggest that between 3.7% and 8.7% are subsequently interpreted incorrectly resulting in a missed fracture.^5–7^ Missed fractures can delay recovery, prolong pain and erode trust in healthcare providers, while incorrectly diagnosed fractures can lead to unnecessary treatments and hospital visits as well as discomfort and inconvenience for patients.^7^ Misdiagnoses increase National Health Service (NHS) costs due to requirements for additional staff, training, insurance and potential litigation.^7^ They can negatively impact clinical staff, causing stress and affecting team morale.^7^

Fractures can occur from infancy to old age and involve any bone. The diverse mechanisms of injury make diagnosis complex and reliant on dedicated imaging expertise.^8^ Yet in practice, the initial evaluation of radiographs for injured patients is often performed by non-specialist clinicians, such as resident doctors or nurse practitioners. These healthcare professionals work in busy and distracting environments, often with inconsistent senior oversight, particularly outside normal working hours or when working in remote services such as minor injury units (MIUs).^9 10^

The accuracy of radiographic interpretive skills varies between and within staff groups, as well as at different times of the day, suggesting that professional experience and working environment are influential factors for those on the clinical frontline.^11 12^ While immediate ‘hot’ reporting by specialist radiographers has previously shown a reduction in diagnostic errors and patients receiving incorrect referral, a shortage of specialist reporting staff makes this solution unfeasible for continuous delivery (24 hours, 7 days) in the NHS.^7 13 14^

Advances in computer vision have led to the development of a range of commercially available artificial intelligence (AI) algorithms designed and approved to assist in fracture detection from plain radiographical imaging, many of which are already in use in hospitals in the UK and globally.^10 15^ These algorithms automatically process radiographs and identify the presence or absence of a fracture, highlighting regions of interest by producing heat maps, boundary boxes or text indicators.^16^ In silico studies have shown high accuracy rates for these tools, suggesting they could potentially reduce misdiagnosed fractures and improve clinical speed and efficiency; however, there is little prospective evidence on their performance in real-world clinical settings.^17 18^ National Institute for Health and Care Excellence (NICE) recommendations outline that certain fracture detection tools demonstrate potential clinical effectiveness and cost-effectiveness for routine clinical use; however, further evidence is needed to justify long-term deployment and widespread funding.^19–23^

To evaluate the impact of introducing AI to assist clinicians in detecting fractures, and its subsequent impact on patients presenting to EDs and MIUs, clinical staff and the wider NHS, we will conduct a multicentre cluster randomised control trial, using a regulatory approved medical device software to assist clinicians in the detection of fractures.

This study aims to directly address a number of the evidence generation recommendations from the NICE EVA. We hypothesise that implementation of an AI fracture detection tool will improve the accuracy of fracture detection, reducing both false positive and negative interpretations compared with contemporaneous ED clinician radiograph interpretation in EDs and MIUs without AI assistance, and will hence reduce the number of unnecessary healthcare contacts such as patient recalls and unnecessary fracture clinic referrals.

### Primary objective

To evaluate whether implementation of an AI fracture detection tool reduces the proportion of patients having unnecessary NHS healthcare contacts resulting from incorrect diagnoses, that is, unnecessary specialist referral or recalls/reattendances.

### Secondary objectives

To assess:

- Diagnostic performance of the AI tool fracture detection tool
  - Comparison of diagnostic performance of AI-assisted fracture detection against the final radiology report as the reference standard.
- Technical performance of the AI fracture detection tool
  - Failure to process images.
  - Turnaround time to provide AI output.
- Impact on clinician experience
  - ED staff user experience and perceived need for senior supervision.
- Patient care experience
  - The proportion of patients receiving inappropriate or omitted appropriate intervention.
  - EQ-5D Health-Related Quality-of-Life Questionnaires
- Service provision
  - The number of ED attendances due to missed fractures or the same injury.
  - ED length of stay.
- Health economic impact of AI-assisted fracture detection
  - Changes in healthcare resource use (ED/MIU attendances, admissions, follow-up imaging, fracture clinic appointments).
  - Cost-effectiveness and budget impact analysis, including quality-adjusted life-years (QALYs), incremental costs, and the overall budget impact of implementing the AI tool within NHS urgent and emergency care pathways.

## Methods and analysis

Systematic Assessment of the Medical Utility of Radiology and Diagnostic Artificial Intelligence (SAMURAI-Fracture) will be a prospective cluster randomised cross-over trial, assessing the impact of an AI fracture detection tool in EDs and MIUs in the UK.

All patients undergoing a plain film radiograph in an ED or associated MIU over a 6-month period for suspected fracture will be eligible for inclusion in the study. Patient identification will be through the routine registration process in EDs and MIUs, where suspected fracture cases are flagged for imaging on electronic patient record (EPR) systems. Clinical and outcome data will be extracted from each site’s EPR and Radiology Information System (RIS)/Picture Archiving and Communication System (PACS), including attendance records, radiology reports, and subsequent ED/fracture clinic activity. Records will be linked at the patient level using NHS number, MRN and X-ray accession number prior to pseudoanonymisation, with the linkage key retained separately from the research dataset by the local site team, as described in the Data de-identification and management section. Data quality will be monitored with a sample of extracted records validated against source EPR/PACS data at each site. Patient-reported outcomes (EQ-5D-5L and experience measures) will be collected via Microsoft Forms, administered to consenting patients within 30 days of their ED/MIU attendance. Patients will be excluded from the study if any of the following apply:

- Patients under 2 years old.
- Skeletal survey conducted to assess for non-accidental injury.
- Skull, facial bone, dental and cervical spine plain film radiographs.
- Thoracolumbar spine plain film radiographs in patients under 18 years old.
- Patient has opted out of data sharing on the National Data Opt-Out.
- Patient completes the opt out form advertised on posters in the ED.

**Figure 1:**
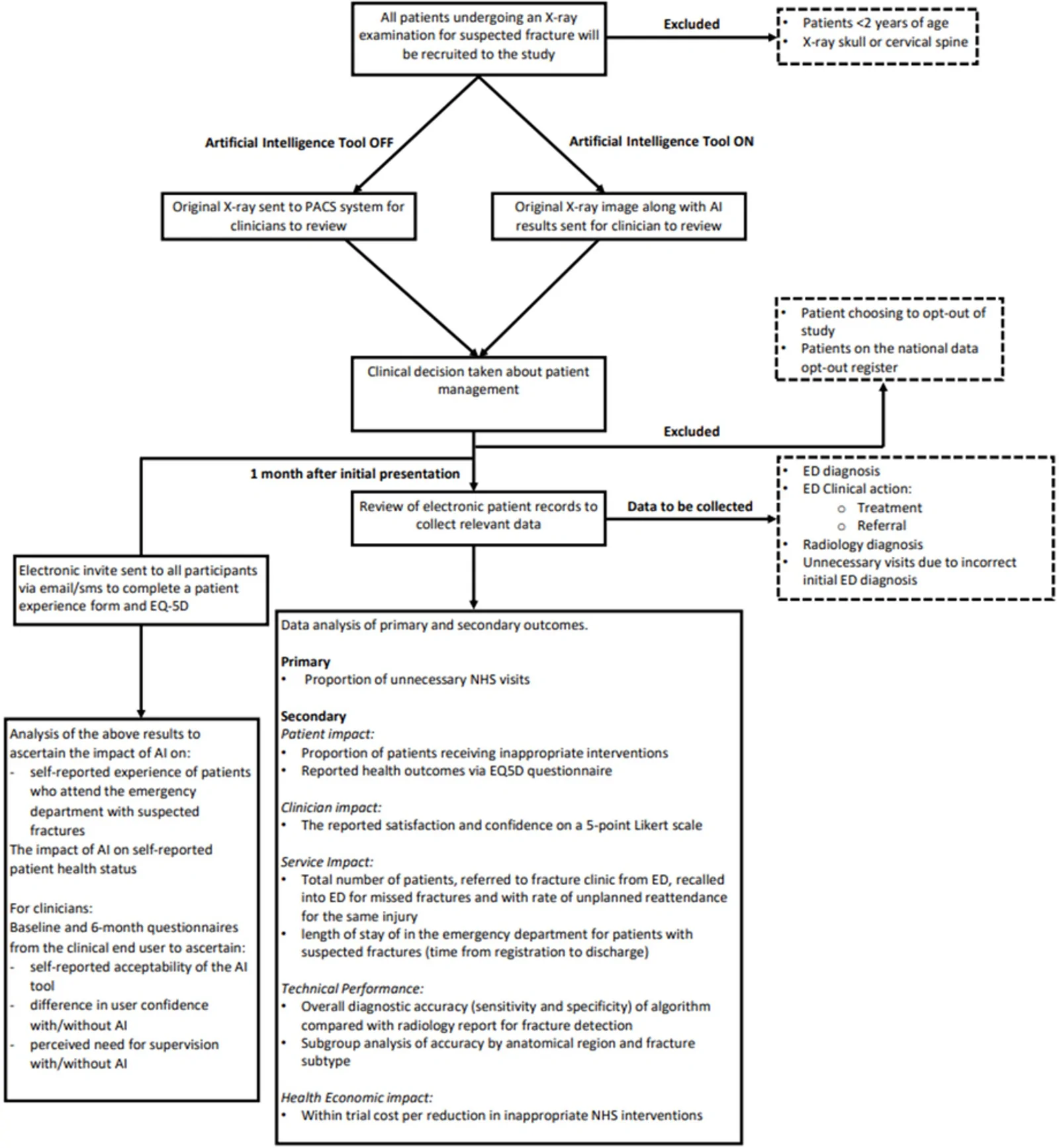
An example output from Radiobotics’ RBfracture algorithm. AI, artificial intelligence; ED, emergency department; NHS, National Health Service; PACS, Picture Archiving and Communication System. EQ-5D: is a short, standardised questionnaire used to measure health-related quality of life.

Figure 1 summarises the overall patient pathway from initial screening to follow-up.

The study uses routinely collected clinical data to evaluate a pathway-level intervention. As the intervention does not alter the clinician’s ultimate management decision or patient outcomes at the point of care, the clinical pathway remains unchanged. Accordingly, identification of eligible patients and collection of outcome data for both primary and secondary endpoints will be conducted under an opt-out consent model. Information about this approach will be communicated through prominently displayed materials in ED and MIU, as well as via the national NHS Data Opt-Out system (copies of these documents are included as online supplemental material).

In addition, a nested subgroup of approximately 30 patients per site will be recruited to complete a brief patient-reported outcome and experience measure (EQ-5D-5L). This subgroup will include patients treated during periods when the AI tool is active (ON) and a comparable group treated when the AI tool is inactive (OFF). Patients will be approached on arrival to the ED and invited to participate, with follow-up completion of the questionnaire at 30 days postattendance. Consent for participation will be documented within the EPR.

Furthermore, approximately 20 clinicians per site will be invited to participate in a qualitative survey assessing perceptions and experiences of AI within their clinical practice. This survey will be administered at both baseline and study completion. Consent information will be clearly presented within the introductory section of each online survey, and completion of the survey will constitute informed consent. Copies of all patient and clinician questionnaires are included in the online supplemental materials.

The four Trusts participating in this study, Oxford University Teaching Hospitals NHS Foundation Trust, Oxford Health NHS Foundation Trust, Royal Berkshire NHS Foundation Trust, and Buckinghamshire Healthcare NHS Trust, are part of a unified imaging network in the Thames Valley. They have been chosen based on their capacity to provide diverse patient populations, encompassing variation in socioeconomic status, geographical location, and ethnicity, as per the INCLUDE guidance, ensuring that results are generalisable to the wider UK population.

### AI software

The AI software used in this study is RBfracture (V.2.4; Radiobotics, Copenhagen, Denmark), a Class IIa CE-marked, AI-enabled medical device designed to support clinicians in the detection of fractures and joint dislocations on plain radiographs in patients aged over 2 years. The software is capable of identifying suspected fractures across the appendicular skeleton, ribs, and thoracolumbar spine, as well as detecting joint effusions (knee and elbow), lipohaemarthrosis of the knee, and periprosthetic fractures.

**Figure 2:**
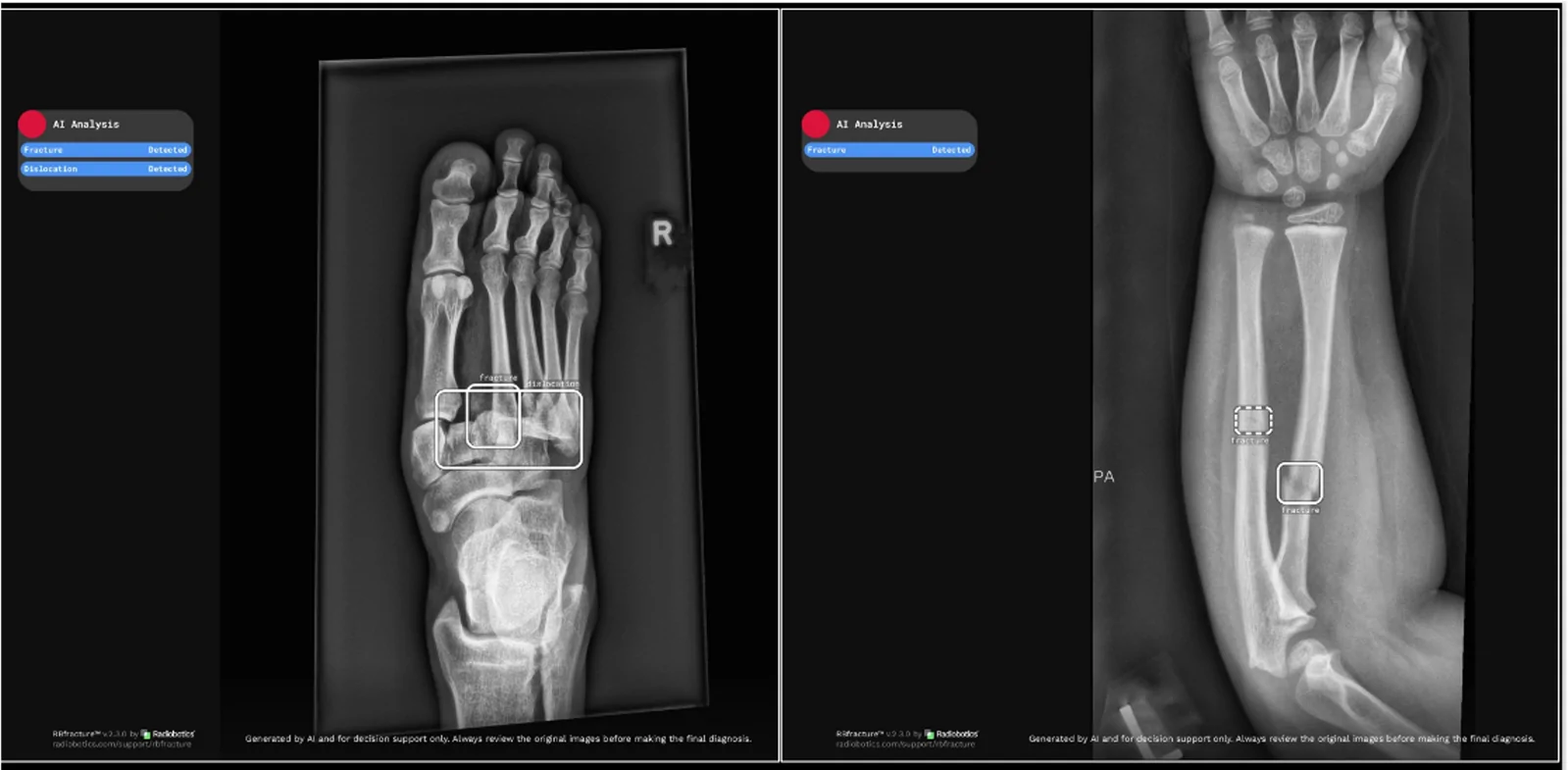
The overall patient pathway from initial screening to follow-up. AI, artificial intelligence. PA: Posteroanterior

RBfracture processes plain radiographs and highlights regions of interest using bounding-box annotations to indicate areas suspicious for pathology. These outputs are intended to support, rather than replace, clinical judgement and do not constitute a definitive diagnosis. The AI-generated annotations are displayed alongside the original images within the standard PACS, allowing seamless integration into the existing clinical workflow without altering image review processes (figure 2). The software version will remain fixed throughout the study period.

### Training and implementation

User training will be delivered in line with the manufacturer’s standard programme, including structured teaching sessions and provision of written guidance on the intended use of RBfracture.

Prior to site activation, clinical staff in ED and MIUs and Radiology will receive standardised training on the RBfracture workflow. This will include instruction on how AI outputs are presented within PACS, clarification of its role as an adjunctive decision-support tool, and its scope of application, including eligible anatomical regions and patient populations. The AI output will be presented as bounding-box overlays within the existing PACS viewer, ensuring no disruption to standard clinical workflows.

Radiobotics will deploy a version-locked instance of the software at each participating site for the duration of the trial. Any required security or infrastructure updates will be documented and communicated to the study team to clearly distinguish these from any changes to the underlying algorithm.

### Operational considerations and monitoring

In the event of software or network downtime, or where radiographs are not processed by the algorithm (eg, due to technical failure or cases falling outside the intended use), standard clinical care pathways will be followed. All such instances will be centrally logged as part of the study’s technical performance evaluation.

Adherence to the allocated intervention status (AI ON/OFF) and clinician interaction with the AI output will be monitored locally by site research teams, with additional oversight provided by PACS teams within each cluster. Any protocol deviations will be reported to the central study team.

### Comparator

The comparator arm will consist of usual clinical practice, defined as contemporaneous interpretation of radiographs in ED and MIU settings without AI-assisted support for suspected fractures.

### Reference standard

The acquired plain radiographs will be subsequently reviewed by a radiologist or reporting radiographer as part of the standard institutional care pathway, with the final verified report acting as the reference standard for comparison against the initial ED clinician’s diagnosis. Reflecting a pragmatic, real-world workflow implementation, formal radiology reporters will not be blinded to the AI algorithm outputs. Discrepant or uncertain cases will not undergo independent research adjudication, as the AI software is utilised strictly as a supportive diagnostic tool rather than an independent decision-maker. Instead, diagnostic or reporting discrepancies will be managed via the normal clinical pathway, with the ultimate decision to treat determined by the responsible treating clinician based on a comprehensive synthesis of the clinical and imaging assessments.

### Cluster randomisation

**Figure 3:**
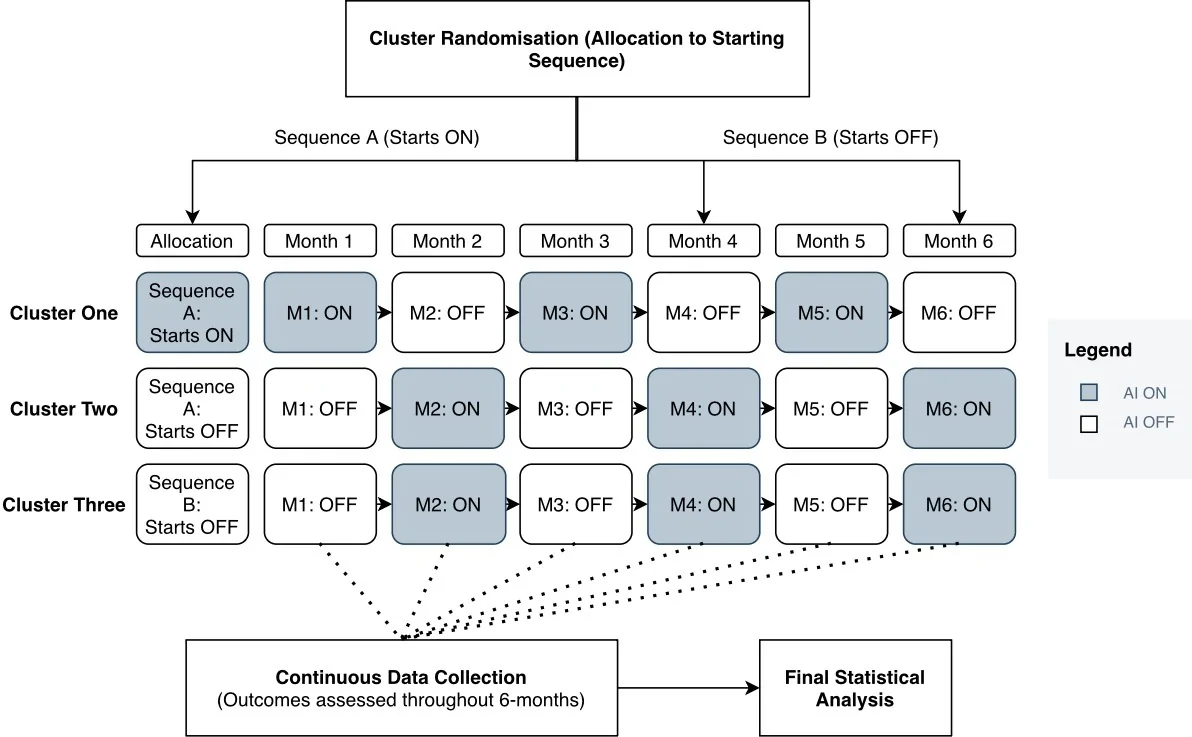
Example sequences of AI-activation following randomisation of clusters. AI, artificial intelligence.

The randomisation sequence for each cluster’s initial AI activation status (AI-on or AI-off in month 1) will be generated by a member of the study team using online randomisation software. A restricted randomisation approach will ensure balance across the three clusters, requiring at least one cluster in each starting condition; allocations assigning all clusters to the same condition will be rejected, and the first valid sequence retained. The sequence will be generated centrally, with code, random seed, and output archived in the Trial Master File to ensure reproducibility and auditability. Allocation will be implemented by the trial statistician, and the core research team will inform all sites so that they can organise locally with their PACS/informatics teams, who will be responsible for activating or deactivating the AI software as appropriate in each changeover. Following initial assignment, each cluster will follow a fixed 6-month alternating schedule (ABABAB/BABABA; figure 3). Data analysis will be undertaken by a statistician who will be blinded as to whether the AI tool was activated or inactivated within a given month.

This study will be divided into three clusters:

- Cluster 1:
  - Oxford University Hospitals NHS Foundation Trust
    - John Radcliffe Hospital ED (level 1 ED).
    - Horton General Hospital ED (level 1 ED).
  - Oxford Health NHS Foundation Trust
    - Abingdon and Witney MIUs (level 3 EDs).
- Cluster 2:
  - Royal Berkshire NHS Foundation Trust
    - Royal Berkshire Hospital ED (level 1 ED).
    - Henley and Townlands MIUs (level 3 ED).
- Cluster 3:
  - Buckinghamshire Healthcare NHS Trust
    - Stoke Mandeville Hospital ED (level 1 ED).
    - Stoke Mandeville MIU (level 3 ED).

Figure 3 illustrates example sequences of AI-activation following randomisation of clusters.

### Outcome measures

The primary outcome is a measure of the proportion of ‘unnecessary healthcare contacts’, a composite measure consisting of unnecessary NHS contact resulting from false negative diagnoses, for example, phone consultations, repeat ED attendance, and those resulting from false positive diagnoses, for example, fracture clinic attendance. This will be recorded as a binary outcome, that is, whether a patient has/has not had an unnecessary healthcare contact during the follow-up period following discharge.

Secondary outcome measures include technical performance of the algorithm, and its impact on service, clinical team, and the patient. Algorithm technical performance will include:

- Measures of diagnostic performance metrics against the final diagnosis provided by the radiology report.
- Subgroup analysis by anatomical region, fracture type, patient demographics, and other existing pathology.
- Number of radiographs not processed by the algorithm that fall under its intended use.
- Patient-reported outcomes via the EQ5D questionnaire.

The major risk from this study includes the calling of false negatives, and false positives from the AI tool. In such cases, clinicians can report specific cases of failures to the central study team. Interim analysis will identify any increases in the false negative rate over the ‘AI-on’ period. Additionally, there is a risk of over-reliance on the tool or errors in interpretation. Clinicians will be reminded to use the AI outputs as an adjunct to their clinical judgement, rather than replacing it. There is a risk of automation bias among clinicians utilising the AI tool, however all radiographs will undergo subsequent interpretation and reporting by a radiologist, reporting radiographer or other trained reporter, with relevant safety-netting protocols in place.

### Statistical analysis

The data analysis plan has been formulated. The study design is a multiperiod treatment crossover design where treatment (the use of an AI tool) is applied sequentially over time to each site. This design has been employed to ensure a balanced distribution of AI on and AI off periods across all sites including longitudinally throughout the progress of the study to minimise the effects of any seasonal variation in injury patterns. Because the real-time presentation of AI bounding boxes may alter baseline clinician interpretive habits, diagnostic modifications could theoretically persist into subsequent control blocks (a learning or carry-over effect). To isolate and control for this potential operational bias, the primary statistical model incorporates an explicit time-dependent carry-over effect term. Within-cluster estimators will separate the direct treatment effects of active AI support from residual behavioural learning shifts across sequential monthly periods. As treatment in one period may affect the response during that period and during a later period (a carryover effect), the comparison between periods with and without the intervention can be biased. To account for any potential bias, we will use a statistical model that allows for within-subject estimators of the carryover and direct treatment effects. A linear model will be used for continuous outcomes and generalised linear models for skewed or categorical outcomes, and survival models where the outcome is time-to-event. Each model will have terms for site, period, treatment and carry-over effects. The level of statistical significance to be used in this study will be α=0.05. Subgroup analyses will explore impact of setting, algorithm, fracture type and age group.

The sample size was calculated to detect an absolute difference of 1.5% in the number of inappropriate NHS contacts. We anticipate an average of 3000 skeletal radiographs for possible fracture per calendar month per Trust. Of these, on average 800 per site are anticipated to show a fracture. Assuming a baseline inappropriate NHS healthcare contact rate of 5.5% (derived from historical data evaluating fracture pathway structural adjustments), a two-sided significance level of 5% is sufficient to detect an absolute reduction to 4.0% in the primary composite outcome of inappropriate healthcare contacts at ~80% power.^24^ Overall, the study will aim to recruit 45 000 patients across all sites. Patient impact will be measured by the total number of cumulative inappropriate contacts made (ie, fracture clinic referrals for false positives, Accidentes & Emergency (A&E) reattendances for false negatives), and patient-reported outcome measures (PROMs) for overall experience of care. Impact on clinician experience of care delivery will be assessed by questionnaire. AI impact on service delivery will be measured by comparing the number of correct and incorrect fracture clinic referrals, fracture related admissions, ED recalls due to missed fractures, ED reattendances and length of stay.

### Patient and public involvement

We have engaged closely with PPI representatives from a previous NIHR-funded study evaluating Assisted fracture detection (FRACT-AI (10.1136/bmjopen-2024-086061)), supported by a dedicated acute healthcare research PPI group at Oxford University Hospitals NHS Foundation Trust. They have been integral in proposal design, including defining our trial outcome measure, as well as improving the language used in the application and Plain English Summary. Participants emphasised this area of research as a high priority and reassured us around the use of retrospective data as proposed in the project outline which they felt was well-justified. Members of the PPI group will join the project Steering Group alongside other PPI members as a specific advisor for the project. We will update the group at key stages of this project and continue to seek PPI engagement and input as we undertake this research. In this project, we will continue to engage patients and users across the involved clinical sites. Evidence will be collected during the prospective phase of the study by approaching staff and patients at each site to complete electronic surveys regarding their experiences of using the algorithm and PROMs, respectively.

Healthcare access challenges often relate to rural isolation, socioeconomic disparities and ethnic diversity.

Underserved ethnicities and vulnerable groups:

RBfracture has been developed on a large, diverse dataset with broad ethnic representation and we continuously investigate and monitor the performance in minority groups.

Geographic and rural barriers:

RBfracture will be deployed within healthcare facilities that do not rely on community-based broadband. The technology can be used locally at urgent treatment centres, minor injuries units and hospitals without requiring consistent internet access for patients.

Economic groups:

RBfracture will not rely on patient-driven data uploads or expensive devices. It will be integrated into NHS systems and made available to all patients through the usual healthcare pathways, ensuring that no additional costs are placed on patients.

Cost of data usage:

Since the system runs on local NHS servers, data processing happens within the secure healthcare network, meaning that patients do not need to use personal data plans or devices to access or benefit from the technology.

Addressing these challenges, RBfracture will focus on the following objectives:

- Reduce healthcare access inequalities by ensuring RBfracture is available at both large urban hospitals and smaller rural health centres.
- Improve diagnostic accuracy in all patient groups, reducing the likelihood of missed fractures, particularly for those in communities that may have less frequent healthcare interactions.
- Target outreach efforts to communities with lower access to healthcare services by collaborating with local NHS Trusts to ensure RBfracture reaches vulnerable groups. The selected sites will allow for sufficient patient heterogeneity (socioeconomic, geographical, population, ethnicity) as per the INCLUDE guidance to ensure that our results are generalisable to the wider UK population.

### Health economic evaluation

A parallel health economic evaluation will be undertaken in accordance with the predefined Health Economic Analysis Plan. The evaluation will assess the cost-effectiveness and budget impact of implementing RBfracture compared with standard care within NHS urgent and emergency care pathways. The primary analysis will be conducted from an NHS and Personal Social Services perspective over a time horizon aligned with the trial follow-up period of approximately 6 months.

A decision-tree model will be developed to represent the fracture detection pathway and the downstream consequences of true positive, true negative, false positive and false negative diagnoses. Model inputs will be informed by trial-derived data on diagnostic accuracy, healthcare resource utilisation, clinical outcomes and EQ-5D-5L patient-reported outcomes, supplemented where necessary by published evidence, expert clinical opinion and national datasets.

Cost categories included in the analysis will comprise RBfracture implementation and licensing costs, ED and MIU attendances, fracture clinic appointments, repeat attendances, hospital admissions, follow-up imaging, treatment associated with missed fractures and other relevant healthcare resource use attributable to the fracture pathway. Unit costs will be obtained from nationally recognised sources, including the NHS National Cost Collection and Personal Social Services Research Unit costs.

Health outcomes will be expressed as QALYs, estimated using EQ-5D-5L responses and the UK value set. Cost-effectiveness outcomes will be reported as incremental costs, incremental QALYs and incremental cost-effectiveness ratios.

A budget impact analysis will also be undertaken to estimate the short-term financial consequences of implementing RBfracture within NHS urgent and emergency care pathways. This analysis will be informed by observed changes in healthcare resource utilisation and the costs associated with implementation of the AI-assisted fracture detection pathway.

Parameter uncertainty will be explored through deterministic and probabilistic sensitivity analyses using appropriate probability distributions for model inputs. Results will be presented using CIs, cost-effectiveness planes and cost-effectiveness acceptability curves, where appropriate.

## Ethics and dissemination

SAMURAI-Fracture received ethical approval from the South Central—Oxford Research Ethics Committee (REC reference: 25/SC/0252, approved 23 October 2025), the UK Health Research Authority (IRAS project ID: 3 57 391-A) and local Research & Development approval from the Joint Research Office at Oxford University Hospitals NHS Foundation Trust (reference: PID18979-SI0013). The study will be conducted in compliance with Good Clinical Practice. Subsequently collected data will be pseudoanonymised and kept among the research team.

The results of SAMURAI-Fracture will be presented at relevant conferences and subsequent publications in peer-reviewed journals will be shared on media platforms to relevant stakeholders and the general public. The full study protocol and statistical analysis plan are available on request to the corresponding author.

## Supplementary material

10.1136/bmjopen-2026-120631 online supplemental file 1

10.1136/bmjopen-2026-120631 online supplemental file 2

10.1136/bmjopen-2026-120631 online supplemental file 3

10.1136/bmjopen-2026-120631 online supplemental file 4
