# Supplementary material for: Systematic assessment of the medical utility of radiology and diagnostic Artificial Intelligence in fracture detection (SAMURAI-fracture): a protocol for a multicentre cluster-randomised controlled trial
Source: BMJ Open. 2026 Aug 21;16(8):e120631. doi: 10.1136/bmjopen-2026-120631 (PMC13504885; doi:10.1136/bmjopen-2026-120631)
Supplement: Supplementary file 1 [file bmjopen-16-8-s001.pdf]

Back

Computer Mobile

SAMURAI-Fracture Study: Baseline Participation Survey

Thank you for completing this survey. This is an essential part of the study and will form part of a longitudinal assessment of the experiences of clinicians in using AI-enhanced imaging. All answers will be confidential, though high-level aggregated data may be shared with industry partners and published in future academic work.

Disclaimer for privacy statement.
The survey is conducted by the SAMURAI-Fracture study research team. Participation is entirely voluntary. You are under no obligation to take part, and choosing not to participate will have no consequences. By completing and submitting the survey, you are giving your informed consent to participate in this study. Recipients of the survey results will be dedicated staff members from the SAMURAI-Fracture study research team.

Any questions or queries, please contact abdala.espinosa@ouh.nhs.uk, or oxcair@ouh.nhs.uk

When you submit this form, it will not automatically collect your details like name and email address unless you provide it yourself.

* Required

1. **What is your full name?** *

Enter your answer

2. **What is your email?** *

Enter your answer

3. **What is your current clinical role or specialty?** *

- Radiologist
