# Supplementary material for: Systematic assessment of the medical utility of radiology and diagnostic Artificial Intelligence in fracture detection (SAMURAI-fracture): a protocol for a multicentre cluster-randomised controlled trial
Source: BMJ Open. 2026 Aug 21;16(8):e120631. doi: 10.1136/bmjopen-2026-120631 (PMC13504885; doi:10.1136/bmjopen-2026-120631)
Supplement: Supplementary file 2 [file bmjopen-16-8-s002.pdf]

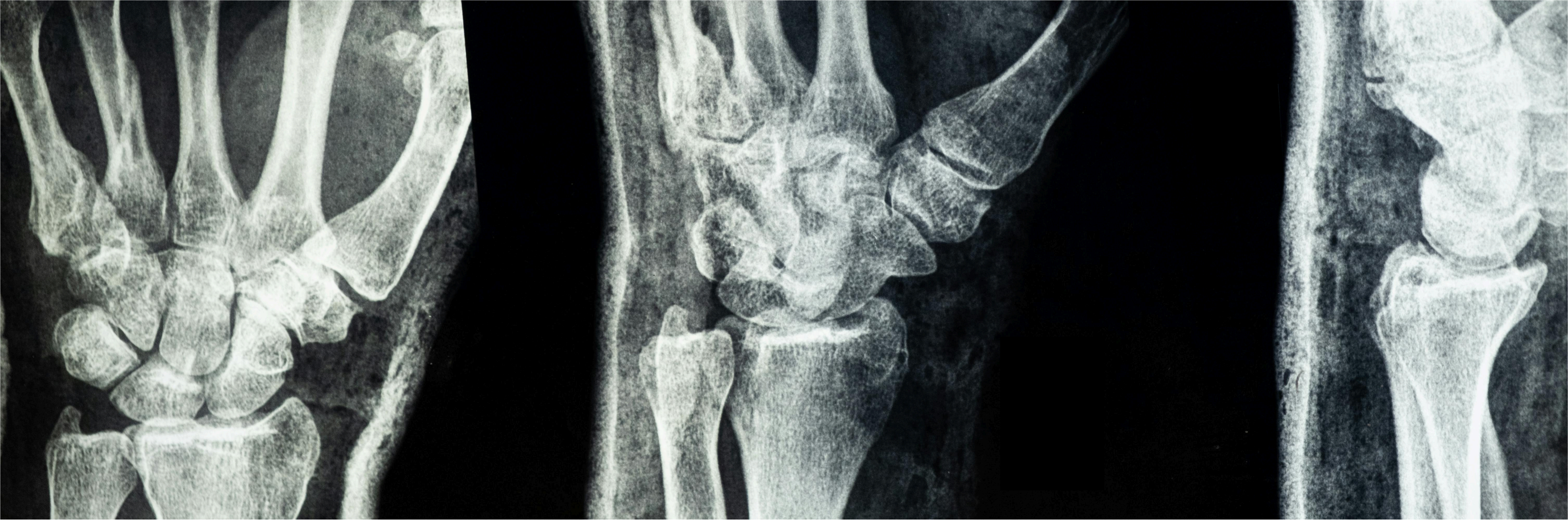

We are testing to see if Artificial Intelligence can help our clinicians spot fractures

We are conducting a research study, called '**SAMURAI Fracture**', to find out if an Artificial Intelligence (AI) software can help reduce the number of missed injuries and unnecessary referrals for patients having an X-Ray for a suspected fracture. The study involves a comparison of X-ray results with and without the help of AI software. **If you are being checked for a possible fracture, you will be entered into the study automatically unless you choose to opt out.**

How does it work?

1. X-ray imaging

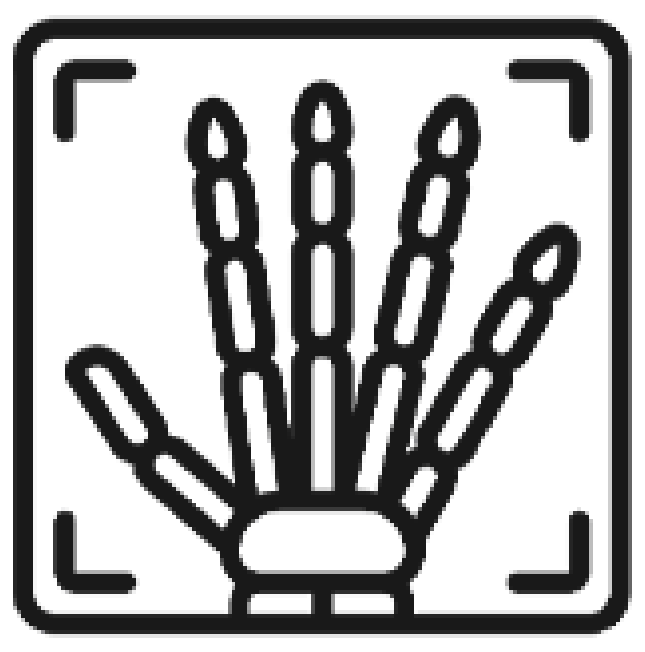

2. Analysis

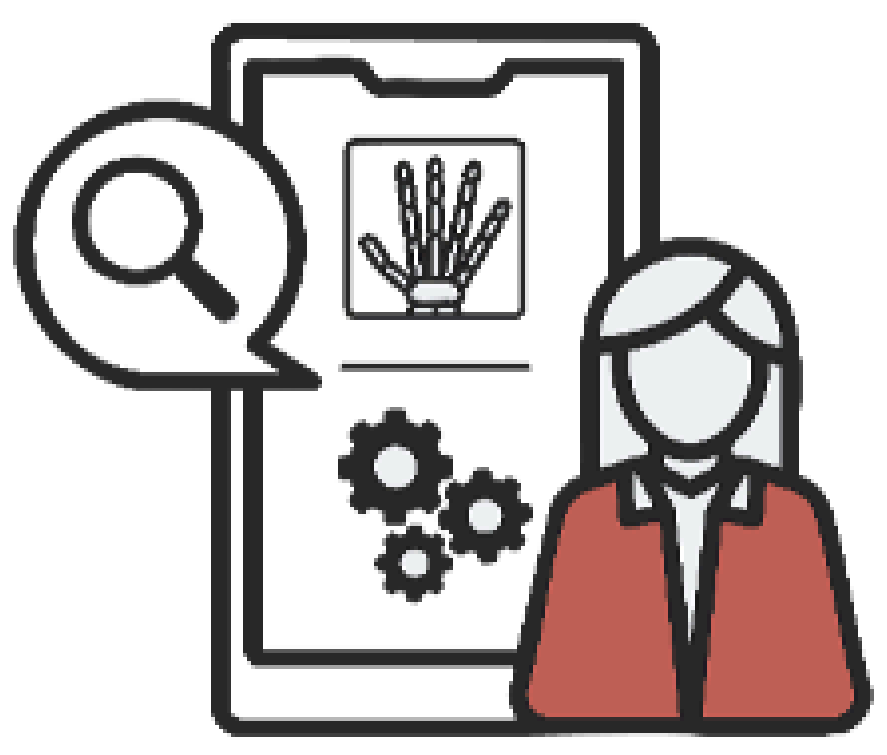

3. Diagnosis*

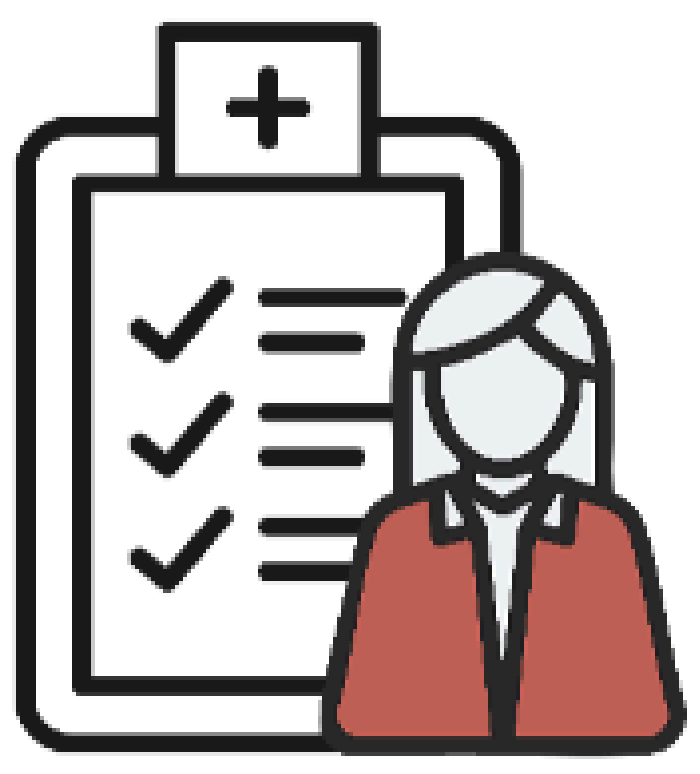

* The decision on patient diagnosis and management is always made by a clinician.

Prefer not to participate?

Your data & privacy are strictly protected and personal details will not be shared with any outside organisations Your anonymised data will be included in the SAMURAI Fracture research study unless you choose to opt out via the following website: **https://digital.nhs.uk/services/national-data-opt-out**

SAMURAI FRACTURE

OxCAIR OXFORD CLINICAL ARTIFICIAL INTELLIGENCE RESEARCH

NHS Oxford University Hospitals NHS Foundation Trust

Powered by Radiobotics®

Version2.0
08.10.2025
