# Supplementary material for: Systematic assessment of the medical utility of radiology and diagnostic Artificial Intelligence in fracture detection (SAMURAI-fracture): a protocol for a multicentre cluster-randomised controlled trial
Source: BMJ Open. 2026 Aug 21;16(8):e120631. doi: 10.1136/bmjopen-2026-120631 (PMC13504885; doi:10.1136/bmjopen-2026-120631)
Supplement: Supplementary file 3 [file bmjopen-16-8-s003.pdf]

Back

Computer Mobile

Health Questionnaire (EQ-5D-5L)

Thank you for taking the time to complete this health questionnaire. It is an important part of the SAMURAI-Fracture study and helps us understand how your health is affected over time. Your answers will be kept confidential, and only grouped, pseudonymised results may be shared with partners or used in future publications.

This questionnaire is conducted by the SAMURAI-Fracture study research team. Taking part is entirely voluntary, and choosing not to take part will not affect your care in any way. By completing and submitting the survey, you are giving your informed consent to participate in this study. Access to the responses will be limited to authorised members of the SAMURAI-Fracture study team.

EQ-5D™, EQ-5D-3L™ and EQ-5D-5L™ are trademarks of the EuroQol Research Foundation. The EQ-5D questionnaire is © EuroQol Research Foundation. All rights reserved. Please do not copy, distribute or modify this questionnaire without permission from the EuroQol Research Foundation.

When you submit this form, it will not automatically collect your details like name and email address unless you provide it yourself.

* Required

Survey Number

1

Please enter the survey number that was given to you by the research team for this questionnaire. This helps us link your answers to the study without using your name or other personal details.
*

Enter your answer

Next
