# Supplementary material for: Systematic assessment of the medical utility of radiology and diagnostic Artificial Intelligence in fracture detection (SAMURAI-fracture): a protocol for a multicentre cluster-randomised controlled trial
Source: BMJ Open. 2026 Aug 21;16(8):e120631. doi: 10.1136/bmjopen-2026-120631 (PMC13504885; doi:10.1136/bmjopen-2026-120631)
Supplement: Supplementary file 4 [file bmjopen-2026-120631.reviewer_comments.pdf]

PEER REVIEW HISTORY

BMJ Open publishes all reviews undertaken for accepted manuscripts. Reviewers are asked to complete a checklist review form and are provided with free text boxes to elaborate on their assessment. These free text comments are reproduced below.

ARTICLE DETAILS

Title (Provisional)

Systematic Assessment of the Medical Utility of Radiology and Diagnostic Artificial Intelligence in Fracture Detection (SAMURAI-Fracture): a Protocol for a Multicentre Cluster-Randomised Controlled Trial

Authors

Novak, Alex; Vaz, James; Espinosa Morgado, Abdala T; Beer , sally; Nash, Katrina; Asif, Ashar; Lundemann, Michael; Metcalfe, David; Costa, Matthew L; Woznitza, Nick; Bajre, Mamta; Kolosnicins, Niks; Clarke, David; Kunnath, Rose; Salem, Ammar; Mandalia, Sachin; Murchison, Andrew G; Shelmerdine, Susan Cheng; Lowe, David; Oke, Jason; Ather, Sarim

---

VERSION 1 - REVIEW

**Reviewer**	**1**
**Name**	**Ye, Shuang**
**Affiliation**	**Shanghai Jiao Tong University School of Medicine, Rheumatology**
**Date**	**11-Jun-2026**
**COI**	**None**

Overall, this is a clinically important and timely study protocol evaluating AI-assisted fracture detection in emergency and minor injury settings. The pragmatic multicentre cluster randomised cross-over design is appropriate in principle, and the planned assessment of diagnostic performance, healthcare resource use, patient experience, clinician experience, and health economic impact is a clear strength.

However, several methodological and reporting issues require clarification before publication.

1.Study status, dates, and registration details

The protocol should clearly state whether the study is planned or ongoing, provide recruitment and follow-up dates, and include trial registration details, protocol version, and access to the statistical analysis plan.

2.Carry-over and learning effects

The authors should provide a clearer justification for the AI-on/AI-off crossover design, including how potential carry-over effects, clinician learning, and behavioural changes between study periods will be handled.

3.Reference standard and diagnostic verification

The final radiology report is used as the reference standard, but the protocol should clarify whether radiology reporters are blinded to AI output and clinical interpretation, and how discrepant or uncertain cases will be adjudicated.

4.Terminology in the sample size section

The protocol states that the study is powered to detect a reduction in the rate of inappropriate NHS interventions from 5.5% to 4.0%, but then refers to this as a reduction in the "missed fracture rate". This should be revised for consistency, as the primary outcome is "inappropriate healthcare contact", not the "missed fracture rate". The authors should use terminology that aligns with the stated primary outcome throughout the sample size justification.

5.Randomisation and allocation procedures

More detail is needed on who will generate the randomization sequence, how allocation will be implemented, whether any stratification or restriction will be used, and how allocation concealment will be maintained.

6.Intervention implementation and fidelity

The protocol should describe the AI workflow in greater detail, including staff training, AI output display, software version control, downtime procedures, adherence monitoring, and handling of images not processed by the algorithm.

7.Data collection and data quality

The authors should specify data sources, linkage methods, outcome data collection processes, quality control procedures, and how patient-reported and clinician-reported outcomes will be collected.

8.Health economic evaluation

The health economic analysis should define the analytical perspective, time horizon, cost categories, model structure, QALY estimation, uncertainty analysis, and budget impact assumptions.

VERSION 1 - AUTHOR RESPONSE

Reviewer: 1

1. Study status, dates, and registration details
The protocol should clearly state whether the study is planned or ongoing, provide recruitment and follow-up dates, and include trial registration details, protocol version, and access to the statistical analysis plan.
Response: Thank you very much. We have completed this information in the protocol as requested.

2. Carry-over and learning effects
The authors should provide a clearer justification for the AI-on/AI-off crossover design, including how potential carry-over effects, clinician learning, and behavioural changes between study periods will be handled.
Response:
We thank the reviewer for this comment. The protocol has been revised to clarify the rationale for the AI-on/AI-off crossover design, which ensures balanced intervention periods across sites and over time to reduce seasonal effects.

We also acknowledge the potential for clinician learning or carry-over effects. To address this, the primary analysis now includes an explicit time-dependent carry-over term, with models incorporating site, period, treatment, and carry-over effects to distinguish direct AI effects from residual behavioural changes.

3. Reference standard and diagnostic verification
The final radiology report is used as the reference standard, but the protocol should clarify whether radiology reporters are blinded to AI output and clinical interpretation, and how discrepant or uncertain cases will be adjudicated.
Response: We thank the editor for this comment. The protocol has been revised to clarify that, consistent with a pragmatic real-world design, radiology reporters will not be blinded to AI output, and the final clinical report will serve as the reference standard.

We also clarify that discrepant or uncertain cases will not undergo independent adjudication but will be managed through standard clinical pathways, with final decisions made by the treating clinician. The protocol now also specifies that outcome assessment will be supported by blinded statistical analysis.

4.Terminology in the sample size section
The protocol states that the study is powered to detect a reduction in the rate of inappropriate NHS interventions from 5.5% to 4.0%, but then refers to this as a reduction in the "missed fracture rate". This should be revised for consistency, as the primary outcome is "inappropriate healthcare contact", not the "missed fracture rate". The authors should use terminology that aligns with the stated primary outcome throughout the sample size justification.
Response: We thank the editor for highlighting this. The protocol has been revised to ensure consistent terminology throughout, with the sample size justification now aligned with the primary outcome of inappropriate healthcare contacts rather than missed fracture rate.

5.Randomisation and allocation procedures

More detail is needed on who will generate the randomization sequence, how allocation will be implemented, whether any stratification or restriction will be used, and how allocation concealment will be maintained.
Response: Thank you. The protocol has been revised to provide additional detail on the randomisation and allocation procedures.

Specifically, the randomisation sequence will be generated centrally using online software with a restricted approach to ensure balance across clusters, and the process (including code and seed) will be archived for reproducibility. Allocation will be implemented by the trial statistician, with sites informed to enact AI activation via local systems. While allocation cannot be concealed operationally due to the nature of the intervention, the statistical analysis will be conducted blinded to allocation status.

6.Intervention implementation and fidelity
The protocol should describe the AI workflow in greater detail, including staff training, AI output display, software version control, downtime procedures, adherence monitoring, and handling of images not processed by the algorithm.
Response: Thank you. We have added a new paragraph describing staff training prior to go-live, how AI output is displayed within the existing PACS workflow, software version control arrangements with Radiobotics for the duration of the trial, the default-to-standard-care procedure for downtime or unprocessed images, and the mechanism used to monitor adherence to correct AI-activation status.

7.Data collection and data quality
The authors should specify data sources, linkage methods, outcome data collection processes, quality control procedures, and how patient-reported and clinician-reported outcomes will be collected.
Response: We thank the editor for this comment. The protocol has been revised to provide detailed information on data sources, linkage, and data collection processes.

Clinical and outcome data will be extracted from EPR and RIS/PACS systems and linked at patient level using NHS number, MRN, and accession number prior to pseudonymisation. Data quality will be ensured through site-level validation of sampled records against source systems. Patient-reported outcomes will be collected via Microsoft Forms within 30 days of attendance, and clinician-reported measures will be captured through structured questionnaires.

8.Health economic evaluation
The health economic analysis should define the analytical perspective, time horizon, cost categories, model structure, QALY estimation, uncertainty analysis, and budget impact assumptions.
Response: We thank the editor for this comment. The protocol has been revised to set out the health economic evaluation in more detail, including the analytical perspective, six-month time horizon, cost categories, model structure, QALY estimation, and uncertainty analysis.

The analysis will be conducted from an NHS and Personal Social Services perspective using a decision-tree model, with QALYs derived from EQ-5D-5L and uncertainty explored through deterministic and probabilistic sensitivity analyses.
