# Supplementary material for: Systematic assessment of the medical utility of radiology and diagnostic Artificial Intelligence in fracture detection (SAMURAI-fracture): a protocol for a multicentre cluster-randomised controlled trial
Source: BMJ Open. 2026 Aug 21;16(8):e120631. doi: 10.1136/bmjopen-2026-120631 (PMC13504885; doi:10.1136/bmjopen-2026-120631)
Supplement: Supplementary file 5 [file bmjopen-16-8-s004.docx]

**SAMURAI-Fracture study: Patient participation survey invitation (via text message)**

Thank you for agreeing to participate in the SAMURAI-Fracture patient experience survey. The survey is conducted by the SAMURAI-Fracture study research team.

**Participation is entirely voluntary.** You are under no obligation to take part, and choosing not to participate will have no consequences.

The survey is intended to be anonymous, and all efforts have been made to ensure anonymity.

Because the survey is anonymous, **once your responses are submitted, they cannot be withdrawn**, as there is no way to link data back to individual respondents.

Link to survey

The responses to the survey are automatically anonymised and aggregated in a manner neither allowing for the identification of individual respondents nor for the attribution of individual responses to a respondent.

The anonymised and aggregated data will be kept for a maximum period of one year after the closure of the survey.

Recipients of the survey results will be dedicated staff members from the SAMURAI-Fracture study research team.

Any questions or queries, please contact [alex.novak@ouh.nhs.uk](mailto:alex.novak@ouh.nhs.uk), or [Teresa.bentabol@ouh.nhs.uk](mailto:Teresa.bentabol@ouh.nhs.uk)
